# Chorionic Bump: Radiologic Features and Pregnancy Outcomes

**DOI:** 10.7759/cureus.11480

**Published:** 2020-11-14

**Authors:** Amman Yousaf, Ahmad Tayyab, Muhammad Sana Ullah Anil, Mohamed Mohamed Helmi Ahmed, Sana Sayed Hussein Badr Ahmed Ahmed, Amal Alobadli

**Affiliations:** 1 Radiology, Hamad General Hospital, Doha, QAT; 2 Radiology, Services Institute of Medical Sciences, Lahore, PAK; 3 Internal Medicine, Services Institute of Medical Sciences, Lahore, PAK; 4 Institute of Public Health Innovation, Washington D.C., USA; 5 Radiology, Hamad Medical Corporation, Doha, QAT; 6 Women's Radiology, Hamad Medical Corporation, Doha, QAT; 7 Women's Radiology, Women's Wellness and Research Center, Clinical Imaging, Hamad Medical Corporation, Doha, QAT

**Keywords:** abortion, first-trimester, ultrasound, chorionic bulge, vaginal bleeding, miscarriage, transvaginal

## Abstract

Background

Chorionic bump is a rare condition defined as a bulge or protrusion from the choriodecidual surface into the gestational sac. The limited literature on this infrequent entity suggests that the pregnancies with multiple chorionic bumps mostly result in fetal demise.

Aims

To review the available literature and the patients from our institute having sonographic findings of chorionic bump and making the sonographers and radiologists aware of this known cause of first-trimester pregnancy loss.

Study design

A retrospective review of the cases diagnosed at our institute during the last four years.

Methods and materials

Single-center institutional data for four years (January 2016-December 2019) was accessed using ICD codes. IRB approval was waived owing to the anonymized use of patient data.

Results

Six female patients diagnosed with chorionic bump were included, with a mean age of 29.83±12 years. The average gestational age at the time of diagnosis was 8.16±3 weeks. The most common sonographic findings were a protrusion from the chorionic wall into the gestational sac cavity, having a central hypoechoic region with peripheral hyperechoic rim (isoechoic to the chorion) and having no vascularity (n=5), and the less common finding was a hyperechoic protrusion with no vascularity (n=1). n=5 had a single lesion, and n=1 had two lesions. The average diameter of the lesion in the largest dimension was 18±11 mm. n=3 pregnancies resulted in a first-trimester miscarriage, and n=3 pregnancies delivered healthy babies at term.

Conclusions

A chorionic bump significantly increases the risk of a first-trimester miscarriage.

## Introduction

Chorionic bump is a rare pathology, often described as a convex polypoid bulge or an ovoid mass protruding from the chorion into the early gestational sac, sometimes seen at the first-trimester ultrasound evaluation [1]. It is typically located within the thickest part of the developing placenta, chorion frondosum [2]. In 2006, Harris et al. first reported this entity by a series of 15 cases with sonographic findings [3]. Numerous sonographic findings can predict the poor pregnancy outcome, i.e., irregular shaped gestational sac (GS), too small or too large GS, a low implantation site, a weak decidual reaction, and a slow fetal heart rate. The literature review suggests that chorionic bump is a cause of first-trimester pregnancy loss, and it doubles the miscarriage rate as compared to when there are no risk factors [3]. In this article, we present a series of six cases of the chorionic bump, which we diagnosed at our institute in the last four years. The sonographic diagnosis was challenging when fetal poles were visible, and a few times, it was misdiagnosed as a subchorionic hematoma or measured as crown-rump length due to the unfamiliarity of this entity among the sonographers and the radiologists. Most of the patients presented with lower abdominal pain, vaginal spotting bleeding, and excessive vomiting and were diagnosed during the first-trimester ultrasound examination that the patients underwent due to these presenting complaints.

This study aims to make the clinicians (radiologists, obstetricians) and sonographers aware of this rare entity, identify the risk factors, describe the sonographic features, and review and correlate with the available literature.

## Materials and methods

Institutional review board (IRB) approval was waived owing to the anonymized use of patient data. The data of four years (from January 2016 to December 2019) was accessed from the archives of the radiology department, Hamad Medical Corporation, using the ICD codes. The term chorionic bump was searched, and a total of six patients were found with the appropriate diagnosis. We believe that it underestimated the incidence because of unawareness of this terminology among sonographers and their misdiagnosis as a subchorionic hematoma. The desired data was collected using Microsoft Excel 2007 (Microsoft Corporation, Redmond, WA). Manual analysis was conducted to make a correlation of chorionic bump with gestational age, mother’s age, number of lesions, and size of lesions, and pregnancy outcome was studied by organizing data on an Excel sheet.

## Results

**Figure 1:**
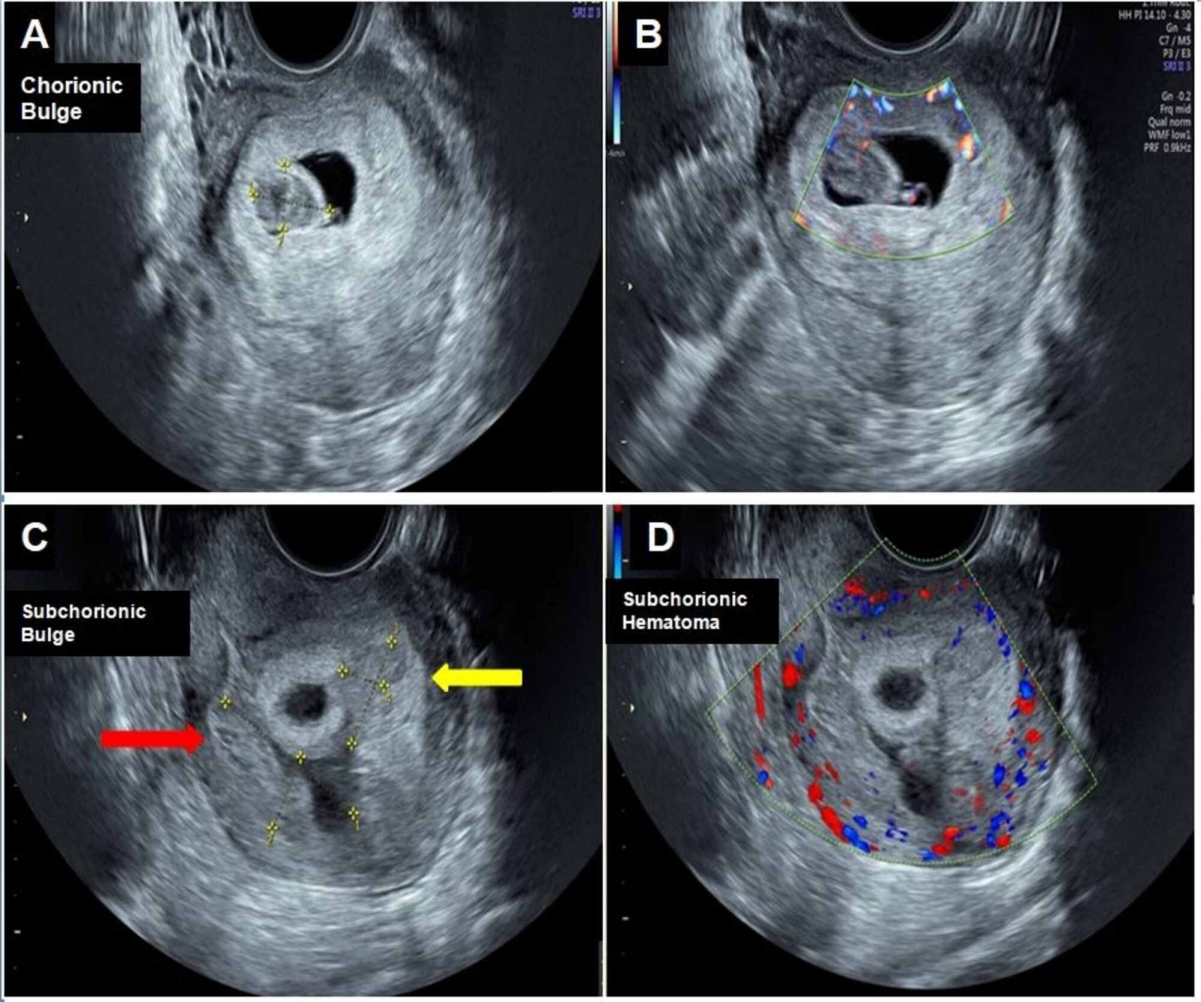
Transvaginal ultrasound (A & B) An oval-shaped bulging lesion from the chorionic wall into the gestational sac, with the hypoechoic center and hyperechoic (isoechoic to chorion) rim measuring 15 X 18 mm in maximum dimensions and showing no blood flow on color Doppler, which is consistent with a chorionic bump (hematoma). (C&D) Two heterogeneous lesions can be seen in the subchorionic area, measuring 2.8 x 1.1 cm (red arrow) and 1.5 x 0.8 cm (yellow arrow), demonstrating no internal flow on color Doppler; they are subchorionic hematomas.

The analysis revealed that out of the six cases presented at our center, three patients (3/6: 50%) gave live births, whereas three patients (3/6: 50%) had missed/incomplete abortions at the time of presentation, and they underwent medical termination of pregnancies. So, it validated the available literature that the chorionic bump increases the likelihood of first-trimester pregnancy loss. The most common sonographic findings were a protrusion from the chorionic wall into the gestational sac cavity, having a central hypoechoic region with peripheral hyperechoic rim, and having no vascularity (n=5) (Figure 1).

**Figure 2:**
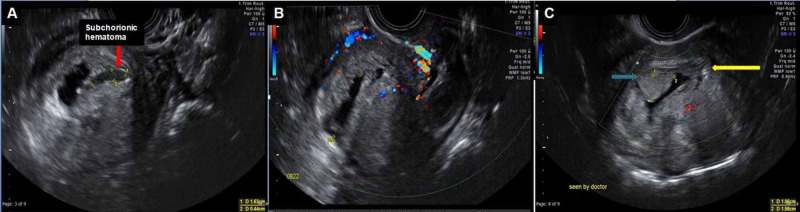
Transvaginal ultrasound during the first trimester (A-C) A distorted low-lying intrauterine gestation sac (yellow arrow) is seen, measuring 28 mm. A subchorionic heterogeneously hypoechoic lesion (red arrow) with no internal vascularity on color Doppler (B) is consistent with subchorionic hematoma. An oval-shaped protrusion from the chorion (isoechoic to the chorion) into the gestational sac, measuring 1 X 1.9 cm, with no vascularity on color Doppler, is suggestive of the chorionic bump.

**Figure 3:**
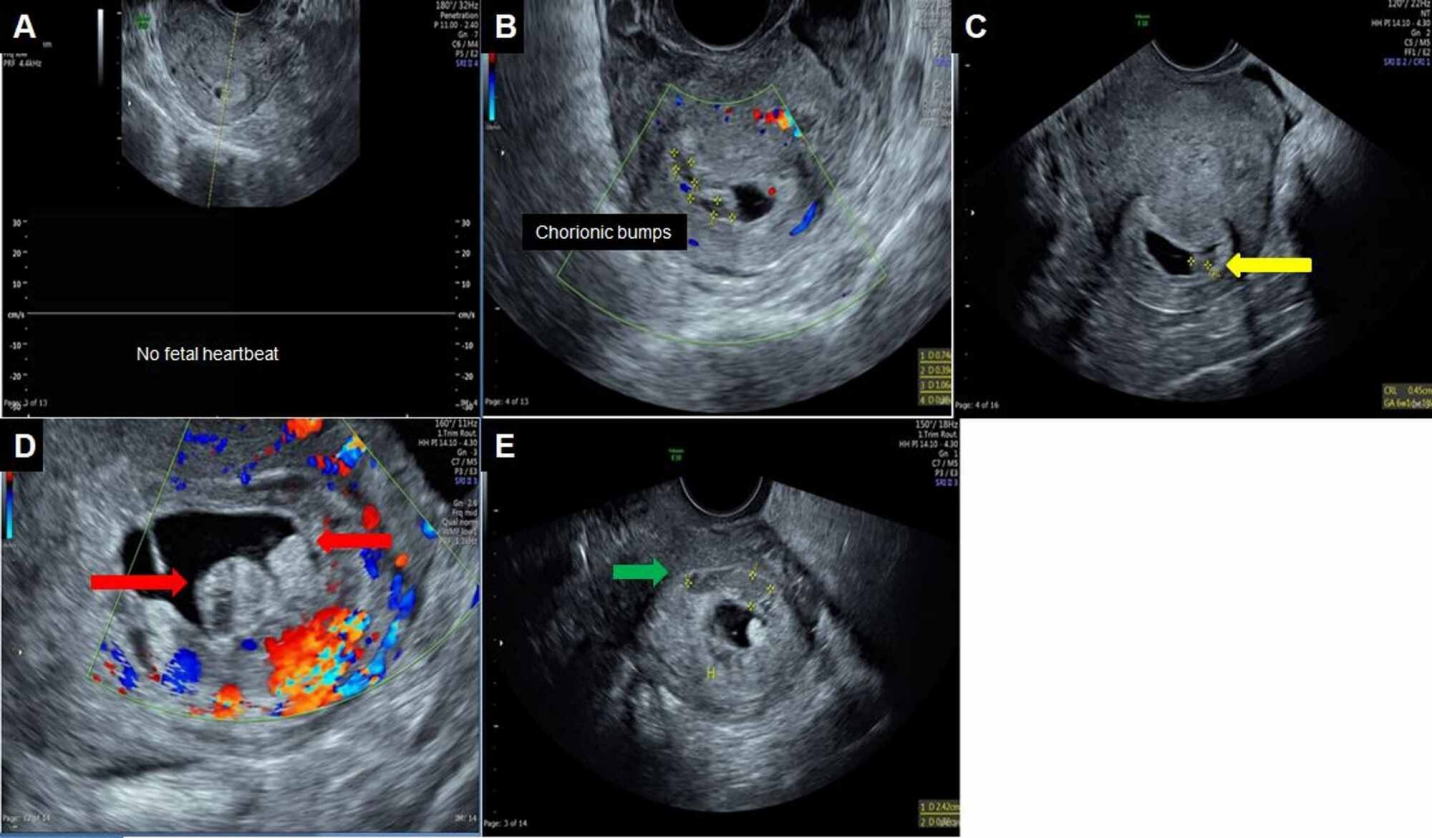
Transvaginal ultrasound (A) The first ultrasound was done at presentation, and it shows a 3 mm gestational sac with no fetal heartbeats. (B & C) There is a small lesion protruding from the gestational sac wall into the cavity, with central hypoechoic and peripheral hyperechoic rim, and no vascularity is consistent with the chorionic bump. (D) A one-month follow-up scan, showing two protrusions from the chorion into the gestational sac with a central hypoechoic and peripheral hyperechoic areas with no vascularity on color Doppler were suggestive of chorionic bumps (these lesions were initially mistaken as gestational sac). (E) A heterogeneously hypoechoic area (green arrow) outside the chorion with no internal vascularity is a subchorionic hematoma.

The less common finding was a hyperechoic protrusion with no vascularity (n=1) (Figure 2).

n=5 had a single lesion, and n=1 had two lesions (Figure 3).

The average diameter of the lesion in the largest dimension was 18±11 mm. Four patients (66.66%) presented with vaginal bleeding or clots and two (33.33%) had lower abdominal pain and excessive vomiting and ultrasound investigation led to the diagnosis of a chorionic bump. The average gestational age at the time of diagnosis was 8.16±3 weeks. Only two patients (33.33%) had a previous abortion history during the first trimester. Four patients (66.66%) were multigravida, and two (33.33%) were primigravida. There were no long-term complications in any of the cases. The summary of the analysis has been presented in Table 1.

**Table 1:** Summary of the analysis FHR; fetal heart rate

Case no.	Patient’s age	Gravida/Para/ Abortus	GA at the time of diagnosis (weeks+ days)	FHR (beats per minute)	No. of chorionic bumps	The largest diameter of chorionic bump (mm)	Sonographic findings of chorionic bump	Pregnancy outcome
1	17	G2P0A1; abortion in the first trimester	5+5	Present	One	15X18	Central hypoechoic, peripheral hyperechoic with no blood flow (Figure 1)	At term delivery
2	37	G2P1A0	7	Absent	One	10X19	Hyperechoic (isoechoic to the chorion), no blood flow (Figure 2)	Incomplete abortion at the time of diagnosis
3	34	G3P1A1	11	Absent	Two	One-10mm Second-6mm	Central hypoechoic with peripheral hyperechoic (isoechoic to the chorion), no blood flow (Figure 3)	Incomplete abortion at the time of diagnosis
4	41	G4P3A0	7+3	Absent	One	16	Central hypoechoic with peripheral hyperechoic (isoechoic to the chorion), no blood flow	Incomplete abortion at the time of diagnosis
5	29	G1P0A0	8	Present	One	14X15	Central hypoechoic with peripheral hyperechoic (isoechoic to the chorion), no blood flow	At term delivery
6	21	G1P0A0	11+4	Present	One	29X15	Central hypoechoic with peripheral hyperechoic (isoechoic to the chorion), no blood flow	At term delivery

## Discussion

A chorionic bump is a convex-shaped irregular bulge that protrudes from the choriodecidual surface into the gestational sac. It is significant to distinguish chorionic bump from subchorionic hemorrhage. The former is the choriodecidual membrane bulging into the gestational sac, whereas the latter is blood collection between chorion and deciduas [4]. It is a rare sonographic finding observed in the first-trimester obstetric ultrasound with a low incidence, from 1.5/1000 to 7/1000 pregnancies [5]. The average gestational age of diagnosis is approximately 11 weeks after the umbilical cord's appearance at the thicker end of the chorion frondosum [4,6]. The exact underlying etiology is unclear. However, the literature hypothesizes that the chorionic bump might be a hematoma, which has been consistently supported by sonographic findings and histopathological analysis. Baalman et al. postulated that the decidualized endometrium's extensive necrosis might be the mechanism behind chorionic bumps [1].

Wax et al. demonstrated that a chorionic bump might be associated with increased risk for fetal aneuploidy and should be a vital counseling point for the patient [7]. The literature also suggests an association between infertility and chorionic bump, though the cause is unknown. A significant association exists between the performance of the ultrasonography (USG) examination for the first trimester bleeding and chorionic bump, giving weight to the theory that the bumps might represent a hematoma [4]. Silva et al. illustrated that abnormal placentation might be correlated with the chorionic bump, further explaining the unwanted maternal (hypertension) and fetal (fetal growth restriction, preterm birth) outcomes [6].

Data on the incidence of miscarriage are controversial. Wax et al. reported that the chorionic bump is associated with a four-time increase in miscarriage incidence. Arleo et al. reported that in otherwise normal pregnancies, the rate of live births was 83% with a single chorionic bump [7-8]. The bump's size or location does not correlate with the pregnancy outcomes; nevertheless, in pregnancies with more than one chorionic bump, the outcome was demise [4]. A prospective study of patients screened at 11-13-week gestation concluded that chorionic bump is an incidental and transient first trimester finding with no bearing on pregnancy outcome. However, this study was limited because it missed the patients presenting earlier than 11 weeks, leading to a somewhat better prognosis [5]. On the contrary, Harris et al. reported a significantly higher prevalence of first and second-trimester pregnancy loss in their study group than matched controls [3]. According to them, the miscarriage rate was twice the normal population and four-fold in patients who underwent assisted reproductive techniques. Sana et al. also reported that the rate of miscarriage in patients with chorionic bumps during the first trimester was double as compared to matched controls [2]. Silva et al. reported a six-fold increase in adverse pregnancy outcomes in patients with chorionic bump identified in first-trimester ultrasound, mainly due to placental mediated diseases [6].

Ultrasonography is the primary imaging modality for the diagnosis. Early diagnosis might suggest the need to follow up with an ultrasound to observe the evolution in the chorionic bump and monitor the pregnancy's viability. The chorionic bump's echogenicity pattern varies, i.e., a central hypoechoic region surrounded by a peripheral hyperechogenic area, entirely hyperechoic, or isoechoic to the chorion [3]. Moreover, on color Doppler, it shows avascularity that favors the chorionic bump to be a hematoma.

The heterogeneous echogenicity, avascularity on ultrasound, and high signal intensity on T1-weighted MR images suggest that the chorionic bump might be a hematoma, further reinforcing hemorrhage finding on the histopathological exam [4]. For two cases, the histopathologic exams showed placental edema and gestational tissue (chorionic villi) [7]. In a case-control study, Silva et al. conducted a uterine artery Doppler in both groups for assessing the risk of abnormal placentation. Although the difference was not statistically significant, a higher value seen in cases might imply a higher risk of placental diseases in patients having a chorionic bump [6].

There is no specific management or guidelines for this condition. It remains unclear whether an intervention might reduce the miscarriage risk in affected patients; however, the chorionic bump might evolve and disappear as the pregnancy progresses [2]. In the various cases reported in the literature, treatment was carried out according to the initial presentation and findings, regardless of a chorionic bump. Nevertheless, it is advisable to keep a close follow-up with serial ultrasounds in the first trimester due to a higher incidence of miscarriage [9].

## Conclusions

This article provides an important clinical review of the association of the chorionic bumps, sonographic features, and pregnancy outcomes. In our study, 50% of the patients (3/6; 50%) had missed/incomplete abortions at the time of presentation during the first trimester, and they underwent medical termination of pregnancies. We can reinforce that the chorionic bump increases the likelihood of first-trimester pregnancy loss. Four patients were multigravida, and two were primigravida, so from this limited data, we can perceive that the chorionic bump is more common in multigravida patients. Radiologists and obstetricians should be aware of this critical finding because of its increase associated with first-trimester pregnancy loss. This article also highlights the need for further research/clinical trials to identify the underlying causative factors and management measures to avoid a first-trimester miscarriage.
